# Innate Intracellular Antiviral Responses Restrict the Amplification of Defective Virus Genomes of Parainfluenza Virus 5

**DOI:** 10.1128/JVI.00246-20

**Published:** 2020-06-16

**Authors:** Elizabeth B. Wignall-Fleming, Andri Vasou, Dan Young, John A. L. Short, David J. Hughes, Steve Goodbourn, Richard E. Randall

**Affiliations:** a School of Biology, Centre for Biomolecular Sciences, University of St. Andrews, St. Andrews, United Kingdom; b Institute for Infection and Immunity, St. George’s, University of London, London, United Kingdom; Instituto de Biotecnologia/UNAM

**Keywords:** defective virus genomes, host cell restriction, innate immunity, paramyxoviruses

## Abstract

During the replication of parainfluenza virus 5 (PIV5), copyback defective virus genomes (DVGs) are erroneously produced and are packaged into “infectious” virus particles. Copyback DVGs are the primary inducers of innate intracellular responses, including the interferon (IFN) response. While DVGs can interfere with the replication of nondefective (ND) virus genomes and activate the IFN-induction cascade before ND PIV5 can block the production of IFN, we demonstrate that the converse is also true, i.e., high levels of ND virus can block the ability of DVGs to activate the IFN-induction cascade. By following the replication and amplification of DVGs in A549 cells that are deficient in a variety of innate intracellular antiviral responses, we show that DVGs induce an uncharacterized IFN-independent innate response(s) that limits their replication. High-throughput sequencing was used to characterize the molecular structure of copyback DVGs. While there appears to be no sequence-specific break or rejoining points for the generation of copyback DVGs, our findings suggest there are region, size, and/or structural preferences selected for during for their amplification.

**IMPORTANCE** Copyback defective virus genomes (DVGs) are powerful inducers of innate immune responses both *in vitro* and *in vivo*. They impact the outcome of natural infections, may help drive virus‐host coevolution, and promote virus persistence. Due to their potent interfering and immunostimulatory properties, DVGs may also be used therapeutically as antivirals and vaccine adjuvants. However, little is known of the host cell restrictions which limit their amplification. We show here that the generation of copyback DVGs readily occurs during parainfluenza virus 5 (PIV5) replication, but that their subsequent amplification is restricted by the induction of innate intracellular responses. Molecular characterization of PIV5 copyback DVGs suggests that while there are no genome sequence-specific breaks or rejoin points for the generation of copyback DVGs, genome region, size, and structural preferences are selected for during their evolution and amplification.

## Abstract (precis)

Copyback defective virus genomes (DVGs) are powerful inducers of innate immune responses both *in vitro* and *in vivo*. They impact the outcome of natural infections, may help drive virus‐host coevolution, and promote virus persistence. Due to their potent interfering and immunostimulatory properties, DVGs may also be used therapeutically as antivirals and vaccine adjuvants. However, little is known of the host cell restrictions which limit their amplification. We show here that the generation of copyback DVGs readily occurs during parainfluenza virus 5 (PIV5) replication, but that their subsequent amplification is restricted by the induction of innate intracellular responses. Molecular characterization of PIV5 copyback DVGs suggests that while there are no genome sequence-specific breaks or rejoin points for the generation of copyback DVGs, genome region, size, and structural preferences are selected for during their evolution and amplification.

## INTRODUCTION

All viruses are prone to replication errors that can lead to the generation of defective viral genomes (DVGs). DVGs have lost at least one essential gene required for replication and, therefore, only replicate in the presence of a standard, nondefective (ND) virus that provides the missing functions. DVGs may also act as “interfering” genomes (defective interfering particles; DIs) that attenuate the replication of the coinfecting standard virus. Advances in molecular techniques have contributed to revealing the role of DVGs in triggering antiviral immunity, and it is rapidly becoming more apparent that DVGs can impact the outcome of natural infections, while driving virus‐host coevolution, and perhaps promoting virus persistence (for reviews on DIs and DVGs see references 1–7). Attention has also been drawn toward the use of DVGs as antivirals and vaccine adjuvants due to their potent interfering and immunostimulatory properties (8, 9). However, the molecular mechanisms that regulate the generation and amplification of DVGs remain largely unknown.

Parainfluenza virus 5 (PIV5; previously referred to as simian virus 5 [SV5]) has a nonsegmented negative-sense RNA genome of 15,246 nucleotides (nt), which encodes eight transcription units (3′-N-V/P-M-F-SH-HN-L-5′), and also carries noncoding leader (Le) and trailer (Tr) sequences at its 3′ and 5′ ends, respectively (for a review on the molecular biology of PIV5 see reference 10 and on paramyxoviruses in general see reference 11). The genome and antigenome are encapsidated by nucleoprotein (NP)-forming viral ribonucleoprotein complexes (RNPs) that protect the viral RNA from degradation, prevent its recognition by the host antiviral responses, and provide the template required for transcription and replication of viral RNA. The virally encoded RNA-dependent RNA polymerase (RdRp) complex recognizes the genomic (Le) promoter elements and drives the expression of viral mRNAs through recognition of *cis*-acting gene start (Gs) and gene end (Ge) elements that encompass each gene. The RdRp also initiates replication of a full-length antigenome from Le. The mechanisms that enable the RdRp to ignore the *cis*-acting elements of the transcription units are not fully understood but are dependent upon the concentration of NP being sufficient to promote the concurrent encapsidation of replicating genomes and antigenomes (12). The encapsidated antigenome acts as the template for genome replication, which is initiated at the antigenomic (Tr) promoter. Le and Tr elements must be in the correct hexamer phase in relation to the encapsidated genomes and antigenomes for RdRp to recognize the encapsidated RNA and initiate virus transcription and replication (13, 14). Initiation of RNA synthesis at the Le and Tr promoters are thought to be mechanistically similar, although the Tr replication promoter is stronger than the Le replication promoter, thereby ensuring more genomes are produced than antigenomes. Efficient viral replication also requires that virus genomes follow the “rule of six,” meaning that the virus genome must be a multiple of six, presumably because 6 nt are associated with one encapsidating NP during the formation of the RNPs (15).

Two major types of DVGs have been described for paramyxoviruses: DVGs that contain internal deletions but retain their 3′ Le and 5′ Tr sequences (16, 17), and trailer copyback DVGs, which maintain an authentic 5′ end terminus and a segment of the viral genome flanked by a reverse complementary version of this segment. In cells in which both ND geneomes and DVGs are replicating, both internal-deletion DVGs and copyback DVGs will have a replicative advantage over ND genomes because of their smaller genome size. Furthermore, because trailer copyback DVGs have a strong (Tr) replication promoter at one end and its complement at the other, it is likely that trailer copyback DVGs will have a replicative advantage over DVGs with internal deletions (18–21). Although, the precise method for the generation of copyback DVGs is not fully understood, the widely accepted mechanism is that copyback DVGs are produced when the viral polymerase detaches from the template and reattaches to the nascent strand, which is then copied back. It has long been thought that the generation of copyback DVGs was a random event generated by the low-fidelity viral polymerases (21). However, the process of DVG generation may not be as stochastic as initially proposed, as previous studies have shown that specific sequences in the genome of vesicular stomatitis virus (VSV) favor the generation of defective RNAs (22). Additionally, a recent study has shown that the generation of DVGs in respiratory syncytial virus (RSV) infections may favor specific regions of the genome, suggesting the existence of hot spots that act as rejoin points for the viral polymerase during the formation of copyback DVGs (23).

Paramyxoviruses are poor activators of early innate immunity for two main reasons. First, they encode IFN antagonists that can both inhibit the activation of the IFN-induction cascade and can block IFN signaling (reviewed in references 24–27). In the case of PIV5, its IFN antagonist, the V protein, interacts with, and blocks the activity of melanoma differentiation-associated protein 5 (MDA5) (28, 29), as well as binding to the protein called laboratory of genetics and physiology 2 (LGP2) to negatively regulate retinoic acid-inducible gene I (RIG-I) (30). In addition, PIV5-V targets STAT1 for proteasome-mediated degradation to block IFN-signaling (31). Paramyxoviruses also tightly control virus transcription and replication, thereby limiting the production of pathogen-associated molecular patterns (PAMPs) that active pathogen recognition receptors (PRRs) and the IFN response (32, 33). However, during replication paramyxoviruses make mistakes, including the generation of copyback DVGs. Copyback DVGs are powerful inducers of innate immune responses both *in vitro* and *in vivo* (18, 19, 34–39). DVG engagement of PRRs activates a number of cellular kinases and transcription factors (e.g., IRF3, NF-κB) that regulate the expression of several cytokines, including interferons (IFNs), tumor necrosis factor (TNF), and interleukin 6 (IL-6) (reviewed in references 40, 41), and can stimulate DC maturation and enhance antigen‐specific immunity to pathogen‐associated antigens (38, 42).

The molecular mechanisms that dictate the generation and accumulation of DVGs remain unknown. Current evidence suggests that both host and viral factors can influence the generation of DVGs. Indeed, the host species and cell type used for virus propagation affect the amplification of DVGs produced by certain viruses, such as influenza viruses and West Nile virus (43, 44). It has also been previously noted that while PIV5 (SV5) DVGs could readily be generated in Vero cells, they could not be generated in MDCK cells (45), although the reason for this was not investigated. Viral factors such as low-fidelity viral polymerases can lead to the overproduction of DVGs due to increased recombination rates (46), while the loss of viral accessory proteins, such as the C protein of Sendai virus, can also promote the accumulation of DVGs (47, 48).

In this study, we show that the generation of copyback DVGs readily occurs during PIV5 replication, but that their subsequent amplification is restricted by their induction of innate intracellular responses. In addition, we used high-throughput sequencing (HTS) to characterize PIV5 copyback DVGs and suggest that while there are no sequence-specific breaks or rejoin points for their generation, size and structural constraints influence their subsequent amplification and evolution.

## RESULTS

### Induction of IFN-β by PIV5.

**FIG 1:**
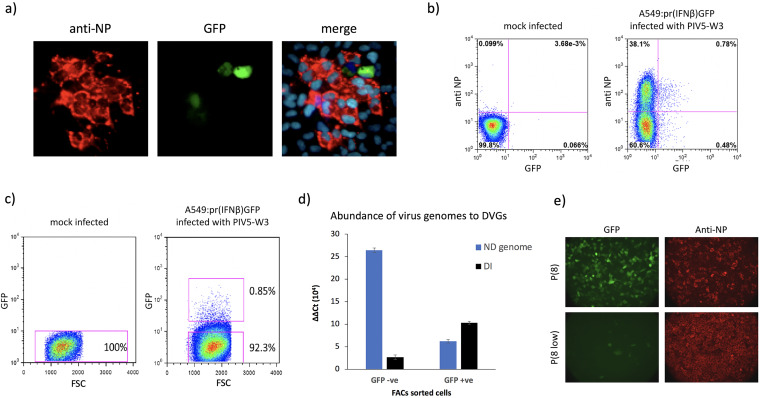
DVGs are enriched in GFP +ve A549/pr(IFN-β)GFP cells infected with PIV5 (wt). (a) A549/pr(IFN-β)GFP reporter cells in which GFP expression is under the IFN-β promoter were grown on coverslips and infected with PIV5-W3 at an MOI of 0.001 PFU/cell. At 2 days p.i. cells were fixed, permeabilized, and stained with an anti-NP monoclonal antibody (red). The nuclei were also visualized by staining the cells with 4′,6-diamidino-2-phenylindole (DAPI) (blue). (b) A549/pr(IFN-β)GFP reporter cells were mock infected or infected with PIV5-W3 at an MOI of 0.001 PFU/cell for 2 days. Cells were trypsinized, fixed, and stained for NP. The percentage of GFP +ve and NP +ve cells in a total cell population of 10,000 cells was determined by flow cytometry using the Beckman Coulter MOFLO (Cytomation) cell sorter (gating lines shown in pink). (c) Flow cytometry of A549/pr(IFN-β)GFP reporter cells that were mock infected or infected with PIV5-W3 at an MOI of 0.0001 PFU/cell for 4 days. Cells were trypsinized and resuspended in 2% fetal calf serum (FCS)/PBS. A population of 10,000 cells was immediately sorted into GFP +ve and GFP −ve cells (gating shown in pink boxes) by flow cytometry using the Beckman Coulter MOFLO (Cytomation) cell sorter. Collected cells were immediately pelleted by centrifugation and the RNA TRIzol extracted prior to real-time quantitative PCR (RT-qPCR). GFP was plotted against cells forward side scatter (FSS). (d) The RNA extracted from GFP −ve and GFP +ve sorted cells (panel c) was subjected to RT-qPCR to determine the relative abundance of genomic RNA to copyback DVG; the ΔC*_T_* values were normalized to the housekeeping gene PPIA. The ΔC*_T_* values were then compared to mock-infected A549/pr(IFN-β)GFP reporter cells to ascertain the ΔΔC*_T_* fold difference. The relative abundance of ND virus genomes and DVGs is shown in blue and black, respectively. (e) Vero cells were infected with the vM8 DVG-rich preparation of PIV5 (W3) (Killip et al. [18]) at low (0.001 PFU/cell) and high (10 PFU/cell) MOIs. At 48 h p.i., the supernatant from these cells was harvested and used to infect A549/pr(IFN-β)GFP reporter cells grown on coverslips. At 24 h p.i., the infected A549/pr(IFN-β)GFP cells were fixed and stained with an anti-NP monoclonal antibody (red).

We have previously shown that during the development of PIV5 (and other negative-sense RNA virus) plaques, only a minority of infected cells are responsible for the production of IFN that induces an antiviral state in the surrounding uninfected cells (34) (Fig. 1a). Furthermore, we, and others, have shown that paramyxovirus DVGs are primary inducers of IFN (18, 19, 34–39). We have suggested that during replication of nondefective (ND) paramyxoviruses (which must initiate virus replication during plaque development), DVGs are produced which subsequently activate the IFN induction cascade in a minority of cells as the virus spreads during plaque development (34). To quantify this, A549/pr(IFN-β)GFP reporter cells (for characterization of this cell line see references 18, 34, and 35) were infected with PIV5-W3 at a multiplicity of infection (MOI) of 0.001 and at 2 days postinfection (p.i.) the cells were trypsinized, fixed, stained for NP and the number of GFP-positive (GFP +ve) cells was compared to the number of cells positive for NP by FACS analysis (Fig. 1b). At this time p.i., the ratio of infected cells in which the IFN-β promoter had not been activated (NP positive; GFP negative cells) to cells in which the IFN-β promoter had been activated (GFP +ve cells) was approximately 30:1.

We next determined whether DVGs were enriched in cells which the IFN-induction cascade had been activated following a low multiplicity spreading infection. A549/pr(IFN-β)GFP cells were infected at an MOI of 0.0001 PFU/cell, with a DVG-poor preparation of PIV5 (to ensure that the likelihood of input DVGs being replicated and amplified was minimal), and at 4 days p.i. the cells were trypsinized and the GFP +ve cells separated from the GFP-negative (GFP −ve) cells by FACS (Fig. 1c). Quantitative PCR was used to estimate the relative abundance of viral genomes and copyback DVGs in the separated populations. DVG primers were designed to be able to detect DVGs that we had previously identified as being produced during passage of PIV5 at high MOI (18). Viral genomes were detected using NP-specific primers in which the primer used for the reverse transcription (RT) step hybridized to genomic RNA. The results showed there was approximately a 5-fold greater abundance of DVGs in the GFP +ve compared to the GFP −ve cells (Fig. 1d). In contrast, there was an approximately 4-fold greater abundance of genomic RNA in the GFP −ve cells compared to the GFP +ve cells.

These observations strongly support the conclusion that during the replication of PIV5, DVGs are rapidly produced and are primarily responsible for the induction of IFN. This conclusion is further supported by the observation that a DVG-rich preparation of wild-type PIV5 [PIV5 (wt)] (vM8) can be “cured” of DVGs by passage at a low MOI, and that the resulting DVG-poor preparation is a poor inducer of IFN (Fig. 1e). (Note: as described in Killip et al. [18], vM designates how many passages a virus has been passaged at high MOI, where sequential preparations are referred to as vM1, vM2, etc.) Nevertheless, the observation that low levels of DVGs can be detected in GFP −ve cells and genomic RNA can be detected in GFP +ve cells suggests that either there may be a dynamic balance within individual cells between the activation of the IFN-induction cascade by DVGs and the ability of ND virus to block its activation, or that in some infected cells the IFN-induction cascade can be activated by PAMPs produced during ND virus replication.

**FIG 2:**
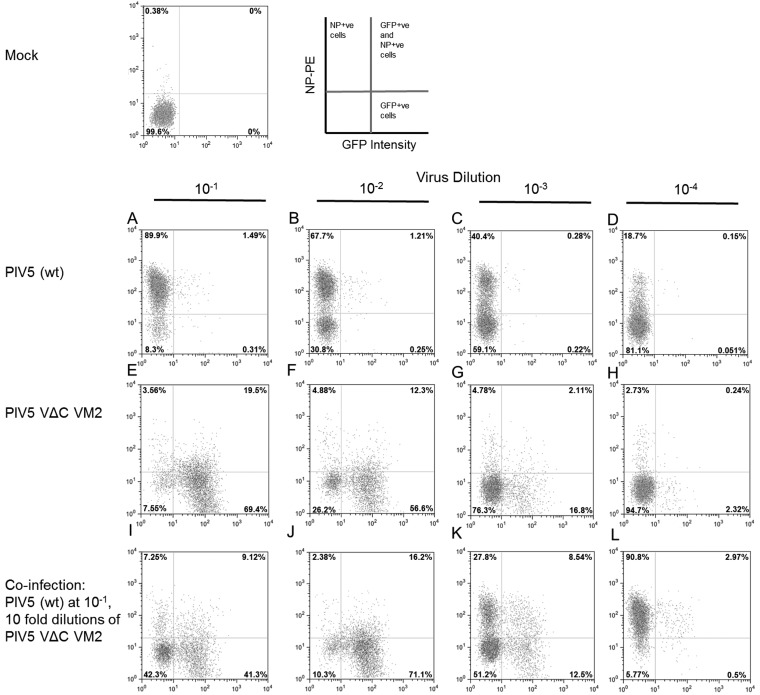
DVGs can block PIV5 replication while inducing the IFN-induction cascade. A549/pr(IFN-β)GFP reporter cells were infected with either PIV5 (wt) (A to D) or PIV5-VΔC vM2 (E to H) at 10-fold dilutions starting at an MOI of 1 PFU/cell. Cells were also coinfected with PIV5 (wt) and PIV5-VΔC vM2 as follows: PIV5 (wt) at 10^−1^ dilution from stock (i.e., 1 PFU/cell), PIV5-VΔC vM2 at 10-fold dilutions of 10^−1^, 10^−2^, 10^−3^, and 10^−4^ (I to L). At 18 h p.i. the cells were trypsinized to a single-cell suspension, fixed, and phycoerythrin (PE)-immunostained for NP. Samples were analyzed by flow cytometry on a Becton, Dickinson FACSCaliber flow cytometer machine. GFP intensity in single cells is shown on the *x*-axis, NP-PE on the *y*-axis.

To further investigate the interaction of ND virus with DVGs and the activation of the IFN response, A549/pr(IFN-β)GFP cells were infected with different dilutions as follows: (i) a DVG-poor preparation PIV5 (wt) (Fig. 2A to D); (ii) a DVG-rich preparation of PIV5-VΔC (vM2; Fig. 2E to H); or (iii) a coinfection with PIV5 (wt) and different dilutions of the DVG-rich preparation of PIV5-VΔC (vM2; Fig. 2I to L). At 18 h p.i. the cells were fixed, immunostained for expression of NP, and the expression of NP (*y*-axis) was plotted against GFP expression (*x*-axis). Of the cells infected with the 10^−1^ dilution of PIV5 (wt), >90% were strongly positive for the expression of NP (Fig. 2A), of which approximately 1.5% were also GFP +ve. In contrast, although 89% of cells were GFP +ve in cells infected with the 10^−1^ dilution of PIV5-VΔC (vM2), a minority of these cells were strongly positive for NP (Fig. 2E). In coinfection experiments, and in agreement with the results of Killip et al. (18), high levels of DVGs did inhibit the expression of NP by PIV5 (wt) in a concentration-dependent manner (Fig. 2I to L). However, although there was some reduction in the number of GFP +ve cells upon coinfection of the 10^−1^ dilution of PIV5-VΔC (vM2) with the 10^−1^ dilution (eqivalent to ∼1 PFU/cell) of PIV5 (wt; compare lanes E and I), PIV5 (wt) did not inhibit the induction of GFP by PIV5-VΔC (vM2) in the majority of cells.

**FIG 3:**
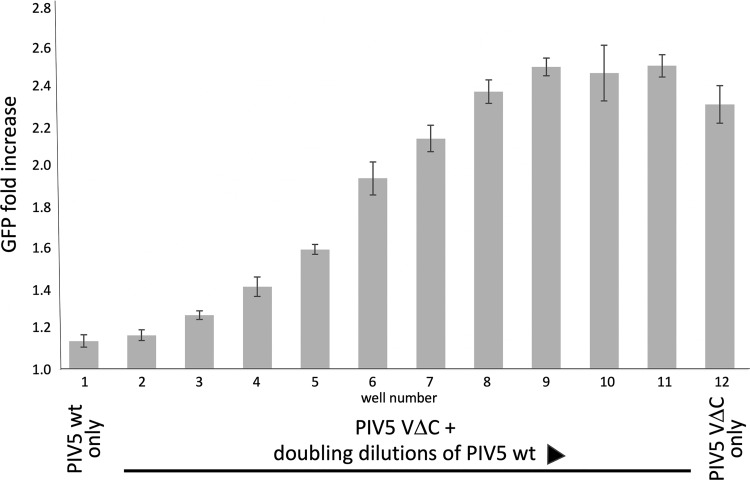
High levels of PIV5 (wt) can block DVG induction of GFP in A549/pr(IFN-β)GFP reporter cells. Monolayers of A549/pr(IFN-β)GFP reporter cells in 96-well microtiter plates were coinfected with a 10^−2^ dilution of PIV5-VΔC vM2 (the highest dilution that still induced GFP expression in ∼80% of cells) and doubling dilutions (wells 2 to 11 in a row of 12 wells) of a DVG-poor stock of PIV5 (wt) starting at a concentration of 200 PFU/cell. No PIV5-VΔC vM2 was added to well 1 and no DVG-poor PIV5 (wt) was added to well 12. At 24 h p.i., the cells were fixed and GFP expression was measured on a Tecan Infinite plate reader and the fold increase, compared to uninfected cells, was calculated. Data shown represents mean values (*n* = 3 replicates; error bars = SD).

Next, to determine whether at higher ratios of ND virus to DVGs the ND virus could block the activation of the IFN-induction cascade by DVGs, A549/pr(IFN-β)GFP reporter cells were coinfected with decreasing amounts of PIV5-W3 (wt) (starting at 200 PFU/cell) and a 10^−2^ dilution of PIV5-VΔC (vM2) that still activated the IFN-induction cascade in the majority of the cells. This experiment clearly showed that at high ratios of ND viruses to DVGs, the ND virus can block DVG activation of the IFN-induction cascade (Fig. 3). Taken together with the experiment presented in Fig. 2, these results suggest that within cells infected with both ND virus and DVGs there will be a balance between the ability of the DVG to induce IFN response and the ND virus to block the response.

### Innate intracellular antiviral responses limit the amplification of DVGs.

Although from the experiments described above it was clear that copyback DVGs are generated during replication of PIV5 in A549 cells, we noted that, in preliminary experiments, we could not produce and maintain DVG-rich virus stocks in these cells, in contrast to Vero cells. We speculated that this may be because DVGs induce interferon and/or other innate intracellular antiviral responses that inhibit their replication in IFN-competent A549 cells. To test this, we passaged PIV5-VΔC in A549 cells that were deficient in a variety of innate responses. We used PIV5-VΔC, rather than PIV5 (wt), in these experiments because we had previously noted that high DVG-rich stocks of PIV5-VΔC could be generated in as little as 2 passages (generating vM2 of PIV5-VΔC) (18) of our working stock of PIV5-VΔC at high MOI in Vero cells. DVG-rich stocks of PIV5-VΔC (vM2) are a mixture of DVG- and ND-viruses, with DVG to ND genome ratios of up to 60:1 (18).

**FIG 4:**
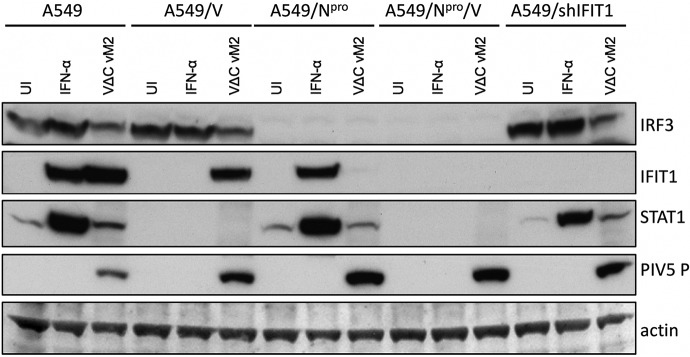
Characterization of A549 and derived cell lines following IFN treatment or infection with a DVG-rich preparation of PIV5-VΔC (vM2). A549, A549/V, A549/N^Pro^, A549/V/N^pro^, and A549/shIFIT1 cells were treated with IFN-β (1,000 IU/ml), infected with PIV5-VΔC vM2, or left untreated (UI). Cell lysates were prepared after 16 h and subjected to immunoblotting for IRF3, IFIT1, STAT1, the P protein of PIV5, and actin. The first 9 lanes of this figure are reprinted from reference 18.

The cell lines used in these experiments were naive A549 cells, A549/V, A549/N^pro^, A549/V/N^pro^, and A549/shIFIT1. A549/V cells cannot respond to IFN as they constitutively express the V protein of PIV5, which targets STAT1 for proteasome-mediated degradation (31). A549/N^pro^ cells cannot produce IFN as they constitutively express N^pro^ from bovine diarrhea virus, which targets IRF-3 for degradation (49). A549/shIFIT1 cells stably express shRNA against *IFIT1*, which is the primary interferon-stimulated gene (ISG) that inhibits PIV5 replication, thereby blocking its expression (50). A549/N^pro^/V cells cannot produce or respond to IFN. In the characterization of these cell lines, as predicted, since IFIT1 expression is upregulated both by IFN and by activated IRF-3, IFIT1 was not upregulated by IFN-β in A549/V cells but was upregulated by the DVG-rich stock of PIV5-VΔC vM2, presumably through the activation of IRF-3. In contrast, IFIT1 was upregulated by IFN-α in A549/N^pro^ cells but was not upregulated by infection of these cells by PIV5-VΔC vM2. IFIT1 was not upregulated by either IFN-α or PIV5-VΔC vM2 in A549/N^pro^/V cells or in A549/shIFIT1 cells (Fig. 4).

**FIG 5:**
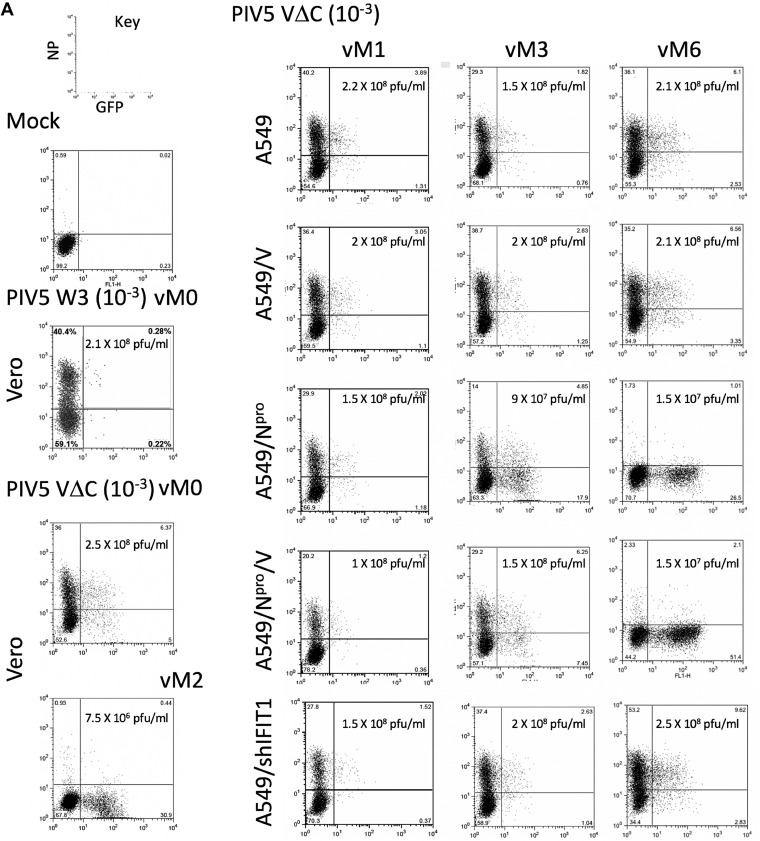

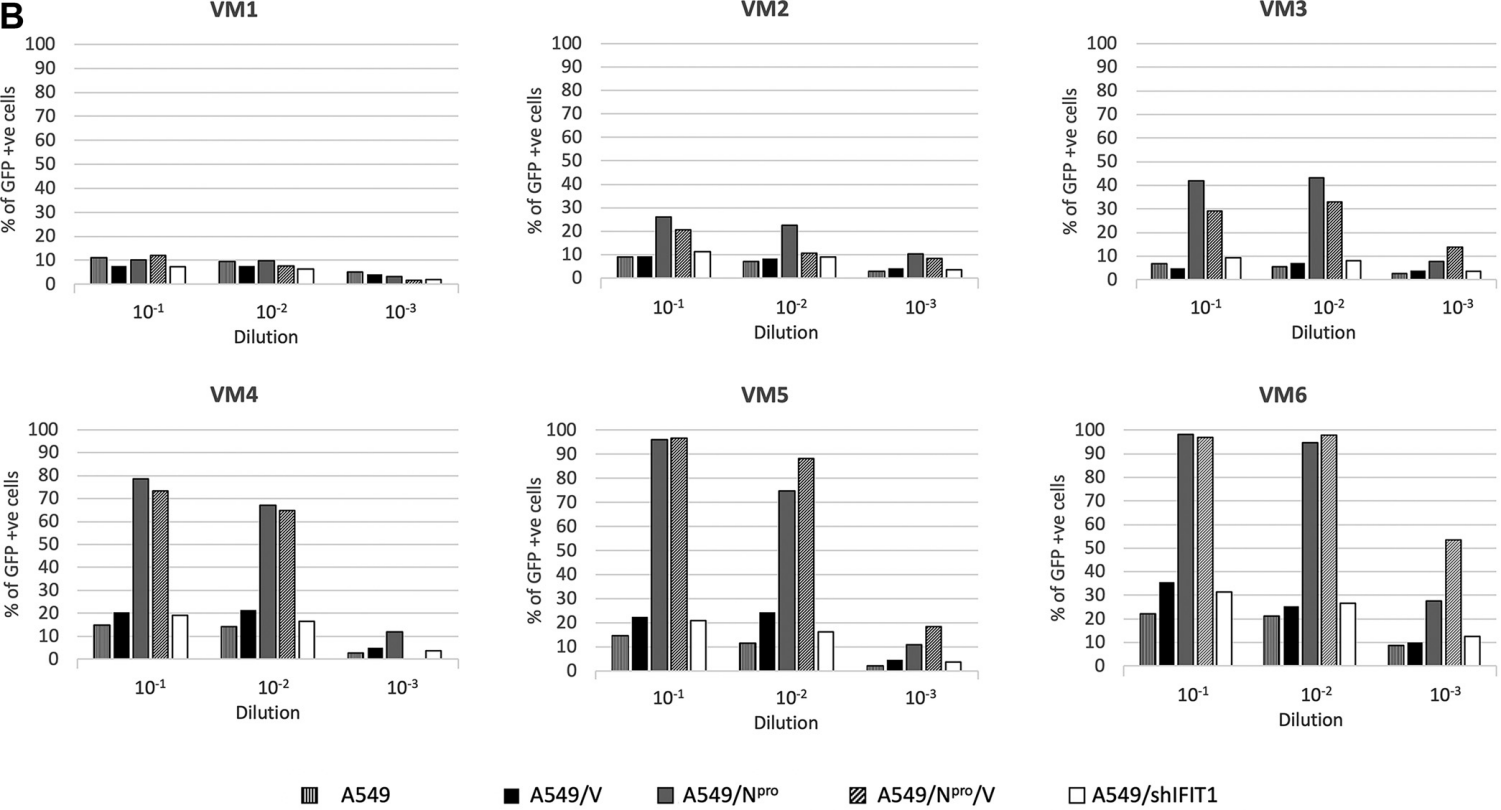
GFP induction in A549/pr(IFN-β)GFP cells following infection with vM1 to vM6 of PIV5-VΔC grown in A549 and derived cell lines. A549/pr(IFN-β)GFP reporter cells were mock infected or infected for 18 h with 10^−3^ dilutions of a DVG-poor stock of PIV5 (wt), vM0 or vM2 stocks of PIV5VΔC prepared in Vero cells, or with a 10^−1^, 10^−2^ or 10^−3^ dilution of the vM1 to vM6 stocks of PIV5VΔC prepared in A549, A549/V, A549/N^pro^, A549/N^pro^/V, or A549/shIFIT1 cells. At 18 h p.i., the cells were trypsinized, fixed, and stained for NP (PE stained). The relative intensity of GFP and PE staining in 10,000 cells was determined by flow cytometry on a Becton, Dickinson FACSCaliber flow cytometer machine. GFP intensity is measured on the *x*-axis, NP-PE on the *y*-axis. (A) Representative plotted graphs of the relative NP and GFP intensity for A549/pr(IFN-β)GFP cells infected with 10^−1^ dilutions of DVG-poor stock of PIV5 (wt), vM0, and vM2 stocks of PIV5VΔC, and a 10^−1^ dilution of the vM1, vM3, and vM6 stocks of PIV5VΔC prepared in A549, A549/V, A549/N^pro^, A549/N^pro^/V, or A549/shIFIT1 cells. Virus titers in these stocks are also shown. (B) The percentage of GFP +ve cells in A549/pr(IFN-β)GFP cells (as determined in panel A) infected with 10^−1^, 10^−2^, and 10^−3^ dilutions of the vM1 to vM6 PIV5VΔC stocks prepared in A549, A549/V, A549/N^pro^, A549/N^pro^/V, or A549/shIFIT1 cells.

These cell lines were sequentially infected six times with PIV5-VΔC at a high MOI (vM1 to vM6) as previously described (18) and samples of the supernatants at each passage were used to infect to A549/pr(IFN-β)GFP reporter cells at different dilutions. At 18 h p.i., infected cells were trypsinized and the percentage of GFP +ve cells (from a sample size of 10,000) was determined by flow cytometry (Fig. 5). Although no significant difference could be seen in the number of GFP +ve cells in cells infected with virus isolated from any of the cell lines after the first passage (vM1), by vM3 the percentage of GFP +ve cells in virus harvested from A549:N^pro^ and A549/N^pro^/V cell lines was significantly higher than from virus harvested from any of the other cell lines. Indeed, by vM4, >60% of the reporter cells were GFP +ve following infection with a 10^−1^ dilution of virus harvested from both the A549/N^pro^ and A549/N^pro^/V expressing cell lines, while only approximately 15% to 20% of cells were GFP +ve using virus passaged in naive A549 cells, A549/V cells, and A549/shIFIT1 cells (Fig. 5b). By vM6 there was an approximate log_10_ reduction in viral titers in the supernatant derived from PIV5-VΔC-infected A549-N^pro^ and A549/N^pro^/V cells (Fig. 5), presumably due to the high levels of DVGs present in these virus preparations (see below).

### Molecular characterization of the DVGs.

**FIG 6:**
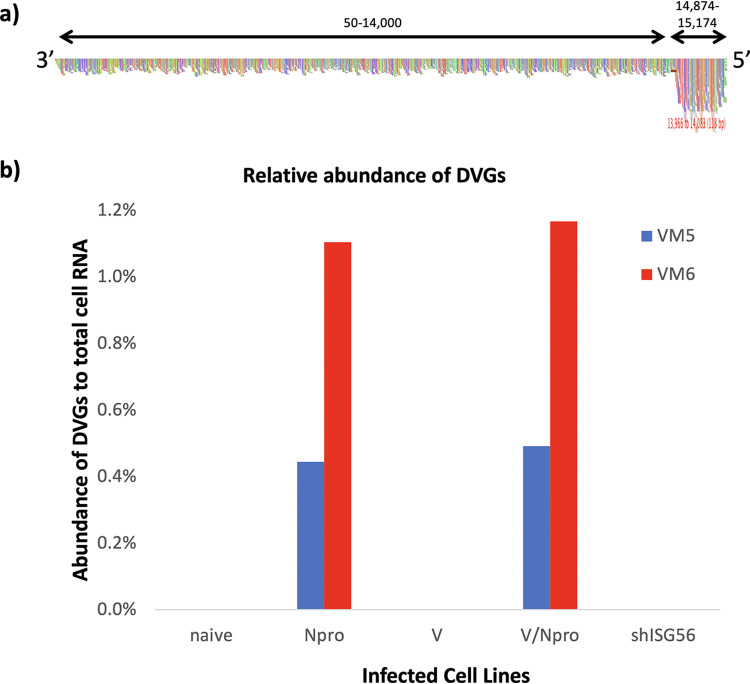
Trailer copyback DVGs in PIV5-VΔC infected cell lines. (a) Nucleocapsids were purified from cells used to generate the vM6 passage series of PIV5-VΔC generated in A549/N^pro^ cells (as also described in Table 1). The reads generated from sequencing were aligned to the PIV5-VΔC reference genome and visualized using Tablet software. The colored vertical lines indicate the read coverage at each nucleotide. Genome positions 1 to 14,000 and 14,874 to 15,174 are indicted by black arrows. (b) The reads generated from DVGs were identified by ViReMa for vM5 and vM6 virus preparations as described in Table 1. The number of DVGs reads was compared to the number of total cellular reads. vM5 and vM6 are shown in blue and red, respectively.

To determine whether the efficiency of activation of the IFN-β promoter correlated with the presence of DVGs, nucleocapsids were purified from cells infected with the vM5 and vM6 virus stocks described above and subjected to high-throughput sequencing as described previously (51). ViReMa software (52) was used to detect and characterize DVGs. Consistent with previous reports (18), reads generated by DVG-rich stocks of virus produce an obvious increase in read coverage at the 5′ end of the genome (e.g., A549/N^pro^ vM5 reads; Fig. 6a). To estimate of the ratio of DVGs to ND virus genomes, the average number of reads per nucleotides (nt) from a region of the genome that was common to all the DVGs (14,874 to 15,174: X) minus the average number of reads per nt prior to the first identified breakpoint (1 to 14,000: Y) was divided by the average number of reads per nt prior the first identified breakpoint (1 to 14,000: Y), i.e., X−Y/Y (Table 1). In addition, the percentage of DVG sequence reads to total cell RNA reads was estimated (Table 1 and Fig. 6b). These data clearly showed there were significantly larger amounts of DVGs in virus stocks made from virus passaged in A549/N^pro^ and A549/N^pro^/V cells than in any of the other cell lines.

**TABLE 1:**
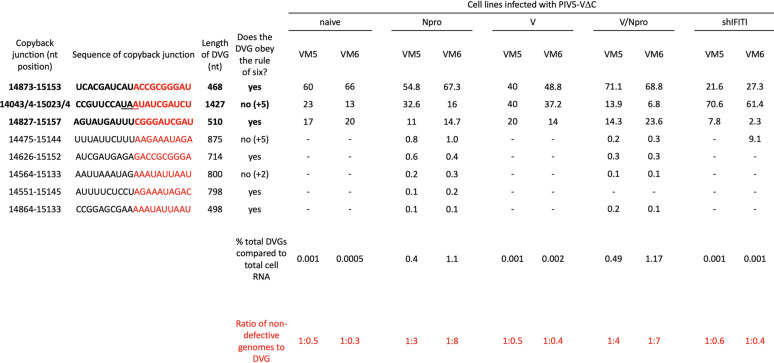
Characterization of the genome structure and relative abundance of copyback DVGs in PIV5-VΔC infected cell lines^*a*^

a Nucleocapsids were purified from cells used to generate the vM5 and vM6 passage series of PIV5-VΔC generated in derivatives of A549 cells and subjected to sequencing. ViReMa software was used to identify breakpoint and rejoin junctions of DVGs. The sequence shown in black indicates the upstream 10-nt antigenome sequence and in red the 10-nt downstream genome sequence of the junctions. Underlined nt indicates a copyback junction where the exact genome position of the copyback junction could not be determined and therefore could be at any of the underlined nt. Also shown are the number of reads generated from DVGs compared to the total cell RNA reads. SAM2CONSENSUS software was used to estimate of the ratio of DVGs to ND virus genomes. The analysis of the HTS data is described in the Results section.

Three major DVGs, first identified by Killip et al. (18) as the most abundant DVGs in a vM2 preparation of PIV5-VΔC prepared in Vero cells, were identified as the most abundant contributors to the total DVG population in all the cell lines tested here (regardless of the relative abundance of DVGs present), except for virus isolated from vM6 infected A549/shIFIT1 cells in which a fourth DVG had become a major contributor to the overall DVG population (Table 1) (Note: the starting vM0 stock used in this study was closely related to that used in the study of Killip et al. [18]). Five additional minor DVGs were also identified; these were almost exclusively found in both N^pro^-expressing cell lines and contributed <1% to the total DVG population. Sequence analysis of the breakpoints (points at which RdRp leaves the antigenome) and reattachment points (where the RdRp attaches to the nascent strand of RNA and continues to process) for each DVG showed no obvious sequence similarities at the copyback junction where the RdRp leaves the antigenome template. However, there did appear to be a 25-nt region of the nascent strand (genome positions 15,133 to 15,157 nt) where the RdRp may preferentially reattach.

To determine whether this 25-nt reattachment region was the same, in an independent experiment in which DVGs were generated, we refined and expanded our analysis of the sequence data on DVGs generated by high MOI passage of PIV5 (wt) in Vero cells previously published by Killip et al. (18). Not only was sequence data available for the vM12 passaged PIV5 (wt), but data were also available for vM8 virus, which was not analyzed in Killip et al. (18). While there were two regions, 14,812 to 14,870 and 15,062 to 15,153 of the nascent strand, that were identified as reattachment points for the majority of DVGs in this passage series, neither of these were the same as the 25-nt reattachment region identified in the PIV5-VΔC passage series.

## DISCUSSION

Following a low multiplicity of infection of cells with DVG-poor preparations of viruses, the chance of an individual cell being infected with both an ND-replicating virus and a DVG that was present in the original virus stock is extremely low. However, we show here that by 2 days postinfection of A549 cells with DVG-poor preparations of PIV5 (wt) at an MOI of 0.001, sufficient numbers of DVGs had been produced to activate the IFN-induction cascade in 0.78% of cells (Fig. 1), demonstrating that the generation of DVGs is a very common event during PIV5 replication. We also show that high levels of ND virus can block the DVG-mediated activation of the IFN-induction cascade, presumably through the IFN antagonism of the V protein or because at high levels of NP the DVG PAMP becomes encapsidated. These data, together with the observation that DVGs can be detected in cells in which the IFN-induction cascade has not been activated, suggest a scenario in which DVGs are generated during virus replication in cells in which the virus has blocked the activation of the IFN-induction cascade. However, as copyback DVGs are packaged into virus particles, “infectious” DVGs released from these cells may activate IRF3 and the IFN-induction cascade in some surrounding uninfected cells before the ND wild-type virus can block this from occurring, thereby explaining why a significant number of GFP +ve cells are negative for NP staining. However, in the experiment in which we infected cells with PIV5 (wt) at a low MOI and measured the percentage of cells positive for NP and GFP (Fig. 1b), approximately 60% of GFP +ve cells (0.78% of the 1.26% of cells that were GFP +ve) were also positive for NP. For technical reasons we could not separate the NP +ve/GFP +ve cells from the NP −ve/GFP +ve cells and probe for the presence or absence of DVGs. It is possible that in these NP +ve/GFP +ve cells DVGs had activated IRF3, but that the subsequent induction of an antiviral state did not occur fast enough for IFIT1 to block virus protein synthesis, while virus replication did not occur quickly enough to block DVG-mediated activation of IRF-3. Alternatively, the IFN-induction cascade may have been activated in a proportion of infected cells by PAMPs produced by virus replication in the absence of DVGs. The latter hypothesis appears to be supported by the observation that during passage of PIV5-VΔC in A549, A549/V, and A549/shIFIT1 cells there was an increase in the number of GFP +ve cells between vM1 and vM6 (Fig. 5) without an obvious increase in the number of DVGs. We are currently trying to distinguish between these two possibilities.

DVG induction of innate intracellular responses limits replication and accumulation of DVGs following high MOI passage. Thus, DVGs accumulated to much higher levels in A549/N^pro^ cells than in naive A549 cells. However, surprisingly, DVGs did not accumulate in A549/V cells or A549/shIFIT1 cells. Furthermore, DVGs did not accumulate to higher levels in A549 cells that constitutively express both N^pro^ and V proteins than in cells that only express N^pro^. These results show that the restriction factor(s) that limits DVG amplification can be induced by DVGs independently of IFN signaling and may be dependent upon IRF-3 activation. However, this factor is not IFIT1, which can be induced by IRF-3 and is the primary ISG that inhibits PIV5 protein synthesis (50, 53). These results are therefore in agreement with those of Tapia et al. (37), which showed that Sendai virus copyback DVGs are generated in the lung of mice independently of type I IFN signaling.

High-throughput sequencing (HTS) was used to determine and characterize the DVGs produced by high MOI passage of PIV5-VΔC in the different cell lines and to determine their relative abundance. No DVGs with different structures to copyback DVGs, including internal deletions, were selected in any of the different cell lines. Furthermore, the most abundant DVGs were similar in all the virus preparations, regardless of the cell line used for the passage series, and were detected in the original vM0 stock of PIV5-VΔC. Thus, it is highly likely that the different DVGs had already been generated by passage of PIV5-VΔC prior to the beginning of this passage series, and that they were or were not amplified during passage, depending on whether or not the cells expressed N^pro^.

It has been suggested for RSV that there are AU-rich hot spot regions that are the rejoin points for RSV copyback DVGs (23). Similarly, for PIV5-VΔC there is a 25-nt-long AU-rich region that may serve as a rejoin point. However, the DVGs generated in an independent passage of PIV5 (wt) did not share this rejoin point. Furthermore, the 4 main rejoin points for PIV5 (wt) DVGs were not particularly AU-rich, having the same content ratio as the rest of the genome. Hexamer phasing is essential for promoter recognition of the RdRp and initiation of virus transcription and replication, and may play a role in RNA editing and influence RdRp disengagement at gene junctions. It may therefore have also influenced where the RdRp disengages from the template antigenome to generate DVGs. However, on analysis, the hexamer phase of the PIV5 (wt) and PIV5-VΔC copyback junctions varied between the DVGs, suggesting that hexamer phasing does not play a role in DVG generation.

From our analysis, there is some suggestion that initially a relatively large copyback DVG may be generated during replication, but on further passage this may further evolve to generate smaller, more efficiently replicated DVGs, that eventually would outcompete the original. Thus, for PIV5 (wt) there are fewer DVGs at vM12 than vM8 and the major DVG (14496–15062) has increased from 87% to 96% (Table 2). Similarly, in the PIV5-VΔC passage series there is a significant reduction in the relative abundance of the largest DVG (14043/4–15023/4) between vM5 and vM6 virus in all the cell lines. Also, although it is clear from this study and that of Murphy et al. (13) that copyback DVGs do not necessarily need to obey the rule of six, the contribution of those that do not decreases during passages of both PIV5 (wt) and PIV5-VΔC. However, there does appear to be a minimum length for the Tr promoter, as no DVG has been identified with <89 nucleotides of the 5′ end (Tables 1 and 2). The requirement for this probably reflects the need to conserve the hexamer phase of the CRII element within the promoter. Since the Tr promoter is found in opposite orientations at both ends, the observation that the minimum size of any DVG identified was 389 nucleotides (which includes 180 nucleotides of both Tr promoters) suggests there may be a minimum optimal size for DVGs and thus for the loop structure. Furthermore, the optimal size for a DVG may be greater than 389 nucleotides, as this small DVG was only a minor population in vM8 preparations of DVG-rich PIV5 (wt) preparations and had been lost by vM12; the major DVG in both vM8 and vM12 preparations was 936 nucleotides long (Table 2). The major DVG in DVG-rich preparations of PIV5-VΔC was 468 nucleotides long (Table 1). Thus, while there appears to be no sequence-specific break or rejoining points for the generation of PIV5 copyback DVGs, there appears to be region, size, and structural preferences selected for during their amplification. However, more detailed studies are needed to fully understand the generation and molecular evolution of DVGs.

**TABLE 2:**
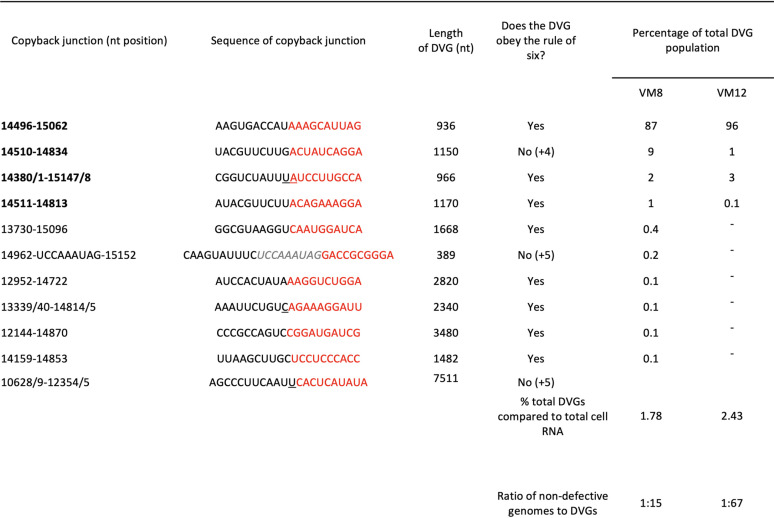
Characterization of the genome structure and relative abundance of copyback DVGs generated in PIV5 (wt)-infected Vero cells^*a*^

a The passage series that was used to generate the vM8 and vM12 RNA used in this analysis has been previously described (18) and the analysis of the HTS data is described in the Results section. The grayed italicized sequence present in the 14962 DVG is an insertion of 9 nt between the identified detachment/reattachment positions.

All viruses produce DVGs during their replication, and these may impact disease outcomes by interfering with the replication of ND virus and by inducing innate immune responses. While the induction of innate antiviral responses may be of major benefit to the host in limiting virus replication, overproduction of DVGs may also overstimulate the immune response, leading to a cytokine storm and increased disease severity. It is therefore of interest that, at least for PIV5, the induction of early intracellular innate responses (that are not induced in cells infected with ND virus) selectively reduces the amplification of DVGs compared to ND virus. Also, the observation that early innate intracellular responses limit the amplification of DVGs suggests that if DVG-rich virus preparations are manufactured for therapeutic purposes, e.g., as vaccine adjuvants, it will be important to use cell lines in which such responses are blocked.

## MATERIALS AND METHODS

### Cells and virus infection.

Vero and A549 cells (obtained from the European Collection of Authenticated Cell Cultures [ECACC]) and A549 derivatives A549/N^pro^ (49), A549/V (18), A549:V/N^pro^, A549:shIFIT1 (50), and A549/pr(IFN-β)GFP (34) were grown as monolayers at 37°C in Dulbecco’s modified Eagle’s medium (DMEM) supplemented with 10% fetal bovine serum. PIV5 wt (strain W3A) and PIV5-VΔC were grown and titrated under appropriate conditions in Vero cells. Virus infections were carried out in DMEM supplemented with 2% fetal bovine serum.

**Immunofluorescence, immunoblot analysis, and fluorescence-activated cell sorting (FACS).** The procedures for immunoblotting and immunofluorescence have previously been described (54). Antibodies used in these procedures included monoclonal antibodies (MAbs) to the phosphoprotein and nucleoproteins of PIV5 (PIV5-Pk, PIV5-NPa, [55]) and rabbit polyclonal antibodies to IFIT1 (Santa Cruz Biotechnology: sc-82946), STAT1 (Santa Cruz Biotechnology: sc-417), and actin (Sigma: A2066). Following immunostaining, monolayers were washed with phosphate-buffered saline (PBS), mounted using Citifluor AF-1 mounting solution (Citifluor Ltd., UK), and examined with a Nikon Microphot-FXA immunofluorescence microscope. For FACS analysis, cells were trypsinized to a single-cell suspension, fixed, and permeabilized as for immunofluorescence, and immunostained with the MAbs to the NP of PIV5. The cells were then incubated with a secondary antibody conjugated to phycoerythrin (PE, Abcam). The percentage of fluorescent cells and the intensity of their fluorescence in 10,000 events were determined using the LYSYS program on a Becton, Dickinson FACScan. Analysis of flow cytometry data was performed using FlowJo software. For live cell sorting, 10,000 A549/pr(IFN-β)GFP reporter cells infected with PIV5-W3 were trypsinized and resuspended in DMEM. GFP intensity was measured against side scatter (SSC). Cells were gated and sorted using the Beckman Coulter MOFLO (cytomation) into GFP +ve and GFP −ve cells and collected in individual vials.

### Real-time quantitative PCR.

RNA was extracted from GFP +ve and GFP −ve cells using TRIzol (Invitrogen) as per the manufacturer’s instructions. cDNA was generated from the extracted RNA using M-MLV reverse transcriptase and virus sequences PCR amplified using GoTaq polymerase (Promega). PCR primers were designed against the virus genome and previously identified large DVGs that had been generated during infection of A549 cells from the same PIV5-VΔC virus stock (18). Oligo-dT primers were also used to generate cDNA to allow normalization of samples to the housekeeping gene PPIA. The cDNA was subject to real-time qPCR performed using SYBR green-based master mix (MESA Blue MasterMix Plus SYBR Assay; Low ROX, Eurogentec). Primer concentrations were optimized for each primer pair. After activation of the polymerase for 5 min at 95°C, the cDNA underwent denaturation for 15 s at 95°C and annealing/extension for 1 min at 60°C for 40 cycles. Real-time qPCR was analyzed by Stratagene Mx3005p thermocycler. Cycle threshold (C*_T_*) values of the uninfected mock cells and the GFP +ve and GFP −ve infected cells were normalized to the housekeeping gene PPIA to ascertain the ΔC*_T_* values. The ΔC*_T_* values of the GFP +ve and GFP −ve infected cells were then compared to those of the uninfected mock cells to determine the ΔΔC*_T_* fold difference.

### Generation of DVG-rich PIV5-VΔC stocks and HTS sequencing of DVGs.

A549 naive, A549/V, A549/N^pro^, A549/V/N^pro^, and A549/shIFIT1 cell lines were infected with PIV5-VΔC (vM0) cells grown in 75-cm^2^ flasks were infected at a multiplicity of infection (MOI) of 5 PFU/cell. The culture medium was harvested every 2 to 3 days; half was frozen at –70°C for subsequent analysis while the other half was used to infect another 75-cm^2^ flask. Sequential preparations of these stocks are referred to as vM1, vM2, etc. RNA was extracted from purified viral RNPs as previously described (18) and sequenced in the Glasgow Polyomics Facility, University of Glasgow, using an Illumina GA2x platform. Reads were aligned to a PIV5-VΔC reference sequence using BWA (56) and visualized using Tablet (57). DVGs were characterized using ViReMa software. The breakpoint junctions of each population of copyback DVG were determined and the number of reads containing the breakpoint were quantified. The contribution of individual DVGs to the total DVG population was determined. The abundance of the total DVG population was compared to the total cell RNA reads. To determine the ratio of DVGs to ND virus genomes, the SAM files generated from the alignment were processed using SAM2CONSENSUS software (https://github.com/vbsreenu/Sam2Consensus) to determine the average coverage of reads at each nucleotide. The average coverage of ND virus genomes (approximately 1 to 14,000 nt that excludes any contribution from reads generated from DVGs) and the average coverage of the reads from a region of genome common to all DVGs (14,874 to 15,246 nt minus the average coverage from nucleotides 1 to 14,000) were compared.
